# Hypersensitive response to *Potato virus Y* in potato cultivar Sárpo Mira is conferred by the *Ny*-*Smira* gene located on the long arm of chromosome IX

**DOI:** 10.1007/s11032-014-0050-2

**Published:** 2014-02-19

**Authors:** Iga Tomczyńska, Florian Jupe, Ingo Hein, Waldemar Marczewski, Jadwiga Śliwka

**Affiliations:** 1 Plant Breeding and Acclimatization Institute-National Research Institute, Młochów Research Centre, Platanowa 19, 05-831 Młochów, Poland; 2 The Sainsbury Laboratory, John Innes Centre, Norwich Research Park, Norwich, NR4 7UH UK; 3 Cell and Molecular Sciences, James Hutton Institute, Dundee, DD2 5DA UK

**Keywords:** Marker, Mapping, MAS, PVY, Resistance, *Solanum tuberosum*

## Abstract

*Potato virus Y* (PVY, *Potyvirus*) is the fifth most important plant virus worldwide in terms of economic and scientific impact. It infects members of the family Solanaceae and causes losses in potato, tomato, tobacco, pepper and petunia production. In potato and its wild relatives, two types of resistance genes against PVY have been identified. While *Ry* genes confer symptomless extreme resistance, *Ny* genes cause a hypersensitive response visible as local necrosis that may also be able to prevent the virus from spreading under certain environmental conditions. The potato cultivar Sárpo Mira originates from Hungary and is highly resistant to PVY, although the source of this resistance remains unknown. We show that cv. Sárpo Mira reacts with a hypersensitive response leading to necrosis after PVY^NTN^ infection in detached leaf, whole plant and grafting assays. The hypersensitivity to PVY^NTN^ segregated amongst 140 individuals of tetraploid progeny of cvs. Sárpo Mira × Maris Piper in a 1:1 ratio, indicating that it was conferred by a single, dominant gene in simplex. Moreover, we identified five DNA markers linked to this trait and located the underlying locus (*Ny*-*Smira*) to the long arm of potato chromosome IX. This position corresponds to the location of the *Ry*_*chc*_ and *Ny*-*1* genes for PVY resistance. A simple PCR marker, located 1 cM from the *Ny*-*Smira* gene, can be recommended for selection of PVY-resistant progeny of cv. Sárpo Mira.

## Introduction

According to the authors, reviewers and editors of *Molecular Plant Pathology*, *Potato virus Y* (PVY, *Potyvirus*) is the fifth most important plant virus worldwide in terms of economic and scientific impact (Scholthof et al. 2011). More than 40 aphid species can transmit the virus in a non-persistent manner to many plant species, mainly from the Solanaceae, where PVY causes crop losses in potato, tomato, tobacco, pepper and petunia production (Scholthof et al. 2011). In potato, the symptoms of PVY infection differ depending on the virus strain and host resistance. The virus strains PVY^O^ and PVY^C^ usually induce mosaic lesions on the leaf, crinkling, necrosis, leaf drop and dwarfing, while PVY^N^ and PVY^NW^ leaf symptoms are hardly visible. In contrast, PVY^NTN^ and some PVY^N^ symptoms can be more severe and include, for example, tuber necrosis (Schubert et al. 2007). Common control strategies for reducing the spread of PVY and thus subsequent crop losses rely on using healthy, certified seed material as well as on growing resistant cultivars. Two types of resistance genes against PVY have been identified in potato and its wild relatives. *Ry* genes confer symptomless extreme resistance (ER), while *Ny* genes cause a hypersensitive reaction (HR) visible as local necrosis that may also be able to prevent virus spreading under certain environmental conditions (Cockerham 1970; Valkonen et al. 1996). As early as 1970, seven *Ny* and five *Ry* genes had been described. Both types were identified in the cultivated potatoes *Solanum tuberosum* ssp. *tuberosum* and ssp. *andigena* as well as in related wild species such as *S. chacoense*, *S. demissum*, *S. hougasii*, *S. microdontum* and *S. stoloniferum* (Cockerham 1970).

To date, six of these genes have been localised in the potato genome. *Ry*_*adg*_ from *S. tuberosum* ssp. *andigena* has been mapped to chromosome XI (Hämäläinen et al. 1997). The second gene, *Ry*_*sto*_ (syn. *Ry*-*f*_*sto*_) originating from *S. stoloniferum*, has been mapped to chromosome XII and is widely present in potato cultivars (Flis et al. 2005; Song et al. 2005; Witek et al. 2006; Zimnoch-Guzowska et al. 2013). Gene *Ry*_*chc*_ has been introgressed from *S. chacoense* into the Japanese potato cultivars (cvs.) Konafubuki and Sakurafubuki and mapped to chromosome IX (Hosaka et al. 2001; Sato et al. 2006). Located at the same locus on chromosome IX is the *Ny*-*1* gene, which confers HR to PVY (Szajko et al. 2008). This gene has been identified in cvs. Rywal, Albatros and Sekwana and shown to effectively prevent the virus from spreading at 20 °C, but not at 28 °C, which is sufficient to provide high field resistance under the average potato growing climatic conditions (Szajko et al. 2008, 2014). Recently, more research into the *Ny*-*1*-mediated reaction to PVY has demonstrated that salicylic acid is crucial for the cv. Rywal resistance (Baebler et al. 2014). *Ny*-*2*, a *Ny* gene conferring HR to PVY both at 20 and 28 °C, has been mapped to chromosome XI in cv. Romula (Szajko et al. 2014). *Ny*_*tbr*_, the first *Ny* gene that was located on the genetic map of potato, resides on chromosome IV and was identified from a diploid backcross population derived from a cross between *S. tuberosum* ssp. *tuberosum* and *S. berthaultii* (Celebi-Toprak et al. 2002).

The importance of breeding for PVY resistance in potato is evident from the fact that all of the genes listed above are currently deployed in commercial breeding efforts and are present in the aforementioned potato cultivars. There are also a number of studies validating and optimising molecular markers linked to these resistances to facilitate marker-assisted selection (MAS), and they are listed below. A multiplex PCR method has been developed for the simultaneous detection of the resistance genes *Ry*-*f*_*sto*_ and *Ns*, conferring resistance to *Potato virus S* (Witek et al. 2006). Similarly, the presence of the *Ry*_*chc*_ gene can be scored in another multiplex PCR together with genes for resistance to potato cyst nematodes, *Potato virus X* and late blight (Mori et al. 2011). Examples of MAS have been published with regard to *Ry*_*adg*_ and/or *Ry*_*sto*_ (Dalla Rizza et al. 2006; Gebhardt et al. 2006;Valkonen et al. 2008; Ottoman et al. 2009). Markers linked to *Ry*_*adg*_ (Kasai et al. 2000) and *Ry*_*sto*_ (Cernák et al. 2008) have been optimised and validated in diverse genetic backgrounds. However, there are some additional PVY-resistant potato cultivars that are of interest to breeders, but for which the underlying genes remain elusive. This includes, for example, the cvs. Santé (Whitworth et al. 2009) and Sárpo Mira.

The potato cv. Sárpo Mira originates from Hungary and its resistance to PVY is described as very high, reaching a score of 9, representing the highest level of resistance on a scale of 1–9 (The European Cultivated Potato Database). To date, the cv. Sárpo Mira has attracted much interest due to its extraordinary resistance to potato late blight, which has been intensively studied (Jupe 2012; Rietman et al. 2012; Orłowska et al. 2012a, b; Tomczyńska et al. 2013). As the pedigree of this cultivar is unknown, the sources of resistance to late blight and PVY remain elusive. It is believed that they might have been introgressed from various *Solanum* species derived from the Vavilov collection, St Petersburg, Russia. Some introgression from *S. demissum* has been demonstrated in the cv. Sárpo Mira in the form of the late blight resistance genes *R3a*, *R3b* and *R4* (Rietman et al. 2012). Despite the ambiguous origin of many resistances, the cv. Sárpo Mira features in several breeding programs and there is thus a need to develop molecular markers linked to the desired disease resistance traits. The potato genome (PGSC 2011) as well as the more recent analysis of the phylogenetic relationship and organisation of potato resistance genes containing nucleotide-binding and leucine-rich repeat domains (NB-LRRs) (Jupe 2012) provide novel tools for identifying markers suitable for MAS (Jupe et al. 2013). In this study we located the PVY resistance gene *Ny*-*Smira* to the long arm of the potato chromosome IX in cv. Sárpo Mira, and we present DNA markers linked to this gene that can be utilised to deploy the resistance in breeding programs.

## Materials and methods

### Plant material

A tetraploid F1 progeny from a cross of the resistant cv. Sárpo Mira and PVY-susceptible Maris Piper (SM × MP) was obtained from the James Hutton Institute, UK. This population has been exploited previously as a mapping population in several late blight resistance studies (Tomczyńska et al. 2013, Jupe 2012). The parental cultivars, a subset of SM × MP progeny (*N* = 140) and cvs. Rywal, containing the *Ny*-*1* gene (Szajko et al. 2008), and Romula, containing the *Ny*-*2* gene (Szajko et al. 2014), as controls, were tested at the Plant Breeding and Acclimatization Institute—National Research Institute, Młochów Research Centre, Poland (IHAR-PIB) for their reaction to PVY, and were genotyped with marker Ry186 (Mori et al. 2011). Individuals from the same F1 family (*N* = 179) were used for genotyping and tetraploid linkage analysis at the James Hutton Institute Sequencing and Microarray facility and BioSS (Jupe 2012).

### PVY resistance assays

The PVY^NTN^ isolate 12-94 (GenBank: AJ889866.1) obtained from potato grown in Poland and kindly provided by the IHAR-PIB Młochów virus collection was used in all resistance tests. This isolate has been described before by Schubert et al. (2007) and Cuevas et al. (2012). The PVY^NTN^ status was verified using the protocol reported by Lorenzen et al. (2006).

Whole plant assays for PVY resistance were performed as described earlier (Chrzanowska 2001; Szajko et al. 2008). Greenhouse-grown, 2–3-week-old plants were transferred to a growth chamber with controlled light (16 h light : 8 h dark) and temperature (20 °C) and, 1 week later, inoculated mechanically with PVY. Inoculated leaves were scored for the presence of necrotic lesions, 4 and 7 days post inoculation (dpi). In 2012, two plants of each cultivar (Sárpo Mira, Maris Piper, Rywal and Romula) were tested by this method at 20 and 28 °C.

In 2012, cvs. Sárpo Mira, Maris Piper, Rywal and 140 individuals of the SM × MP progeny were tested in detached leaf assays as described by Szajko et al. (2008). The experiment was repeated on two dates and three leaves per genotype were inoculated each time. Leaves were incubated at 20 °C in high humidity (nearly 100 %). HR appeared from 4 to 7 dpi and their presence or absence was scored visually twice, at 4 and 7 dpi.

In order to increase infection pressure, we also inoculated cv. Sárpo Mira by grafting scions of PVY-infected tobacco shoots on cv. Sárpo Mira rootstocks as described by Chrzanowska (2001). In 2012, six plants of cv. Sárpo Mira were inoculated by grafting in the greenhouse and 4 weeks later new potato shoots growing under the point of grafting were scored for the presence of HR symptoms.

In 2013, a whole plant assay was performed on cvs. Sárpo Mira, Maris Piper, Rywal and 140 individuals of the SM × MP progeny. Three plants per genotype were tested at 20 °C. Three weeks after inoculation, fully expanded top leaves from the tested cultivars and 17 SM × MP individuals for which opposing results were obtained in the detached leaf and the whole plant assay were collected in three replicates and tested for the presence of PVY by an ELISA using a cocktail of monoclonal antibodies (PVY-mono-cock, Bioreba AG, Switzerland).

### DNA extraction and marker Ry186 scoring

Genomic DNA was extracted from 1 g of fresh young leaves of 140 genotypes of SM × MP progeny and two parent plants grown in the greenhouse, using the DNeasy Plant Maxi kit (Qiagen, Hilden, Germany) according to the manufacturer’s instructions. Marker Ry186 was amplified as described by Mori et al. (2011) with the following modifications to the procedure: the reaction mixture of 20 μl contained 2 μl of 10 × Taq PCR buffer Mg^2+^ Plus, the four deoxynucleotides (0.1 mM; Sigma-Aldrich, St. Louis, MO, USA), primers (F: TGGTAGGGATATTTTCCTTAGA, R: GCAAATCCTAGGTTATCAACTCA) (0.2 μM; Sigma-Aldrich, St. Louis, MO, USA), *Taq* DNA polymerase (0.05 U/μl; GenoPlast Biochemicals, Pruszków, Poland) and 10–30 ng of template DNA. The PCR program conditions were: 94 °C for 180 s; 39 cycles of 94 °C for 30 s, 55 °C for 45 s and 72 °C for 60 s; followed by a final extension at 72 °C for 420 s. PCR products were separated by electrophoresis in 1.5 % agarose gels stained with ethidium bromide and visualised on a ultraviolet transilluminator.

### Genotyping by GoldenGate assay (Illumina) and tetraploid map construction

The experiment was carried out by the Sequencing and Microarray facility at the James Hutton Institute as described in Jupe (2012). Briefly, 176 individual F1 SM × MP plants and parents were tested for 1152 SNP markers derived from alignments of EST sequences of three US potato cultivars with the DM genome sequence, in an Illumina GoldenGate assay.

### Data analyses

A Chi squared test was applied to compare the obtained and expected segregation ratios using the computer program STATISTICA for Windows (Stat Soft, Inc., Tulsa, OK, USA). A map of a single linkage group of chromosome IX was constructed using only simplex GoldenGate markers and Ry186 linked to the PVY resistance in coupling. The mapping population consisted of 112 SM × MP individuals for which we had both PVY resistance phenotypes and GoldenGate genotyping data available. Linkage analysis was performed using JoinMap^®^ 4 (Van Ooijen 2006) with the following settings: CP population type [“a population resulting from a cross between two heterogeneously heterozygous and homozygous diploid parents, linkage phases originally (possibly) unknown” (Van Ooijen 2006)], independence of LOD as a grouping parameter (linkages with LOD > 3 were considered significant), regression mapping algorithm and Haldane’s mapping function. Map comparisons were carried out using the “combine maps” function of the JoinMap 4 software (Van Ooijen 2006).

### NB-LRR gene cluster analysis

To identify the potential *R* gene locus underpinning the resistance, positions for markers Ry364, TG328 and 38-510 (Sato et al. 2006) in the potato genome were retrieved by applying BLASTn searches against the sequenced potato reference clone DM pseudomolecules version 4.03 (Sharma et al. 2013; http://solanaceae.plantbiology.msu.edu/pgsc_download.shtml). The retrieved positions were compared to the potato NB-LRR gene cluster positions detailed by Jupe et al. (2013).

## Results

**Fig. 1:**
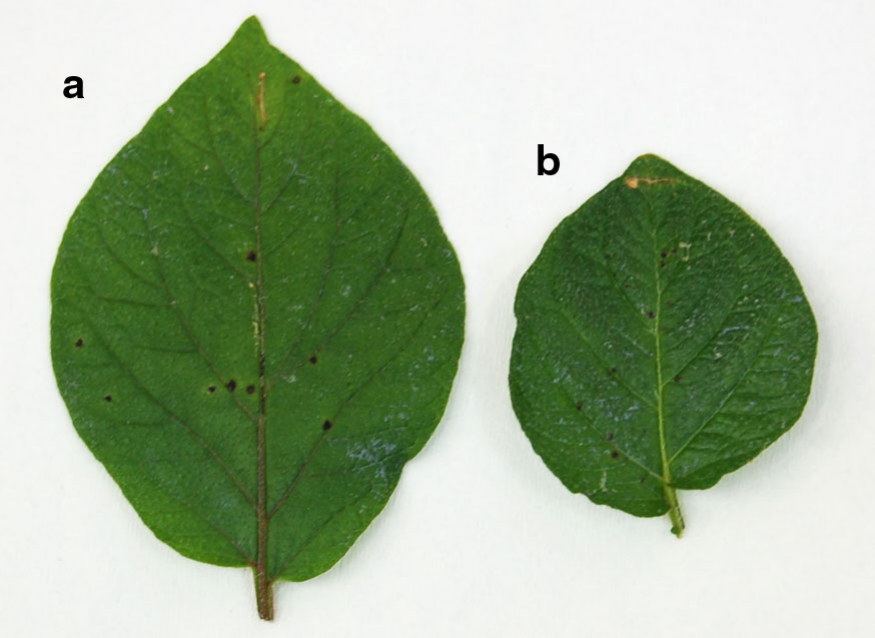
Necrotic lesions symptomatic for a hypersensitive response to PVY^NTN^ on cv. Sárpo Mira (**a**) and cv. Rywal (**b**). Leaves cut from a whole plant, test carried out at 20 °C

PVY^NTN^ infection in the cultivar Sárpo Mira yielded a hypersensitive response in detached leaf assays, after grafting, and in whole plant assays, while cv. Maris Piper did not exhibit such cell death response in detached leaf or whole plant assays (Table 1). Sárpo Mira and cultivar Romula gave HR at 20 and 28 °C whereas the cv. Rywal only yielded HR at 20 °C. Typical responses of cvs. Sárpo Mira and Rywal are shown in Fig. 1.

**Table 1:** Assessment of hypersensitive response (HR) to PVY^NTN^ infection in parents of the mapping population (cvs. Sárpo Mira and Maris Piper) and standards [cvs. Rywal and Romula (Szajko et al. 2008, 2014)]

Cultivar	HR to PVY in			
	Whole plant test, 20 °C	Whole plant test, 28 °C	Grafting test (2012), greenhouse conditions	Detached leaf test, 20 °C
Sárpo Mira	1	1	1	1
Maris Piper	0	0	nt	0
Rywal (*Ny*-*1*)	1	0	nt	1
Romula (*Ny*-*2*)	1	1	nt	nt

1—Present, 0—absent

*nt* Not tested

Cultivar Sárpo Mira also showed HR symptoms under the strong infection pressure in a grafting assay, where PVY-infected tobacco shoots were grafted on cv. Sárpo Mira rootstocks under greenhouse conditions. Significant temperature fluctuations were measured in the greenhouse, where the temperature could reach 30 °C and above. HR started to appear on new shoots of cv. Sárpo Mira 3 weeks after grafting.

In order to test the inheritance of the ability to induce HR to PVY infection, 140 F1 progeny of the SM × MP cross were assessed in detached leaf assays in 2012 (Table 2). High humidity induced the formation of calli on the leaf surface, which made the observation of the reaction difficult in some genotypes in these tests. Thus, in 2013 whole plant tests were performed on parental cultivars, standards and individuals from the mapping population (Table 2). The results were consistent between the assays for both the parental cultivars, cv. Rywal and the majority of the mapping population (123 individuals). While 62 progeny individuals reacted with HR to PVY inoculation, 61 did not show any symptoms in either test. However, the results of these two tests were contradictory for 17 progeny genotypes. These 17 plants, as well as plants of cvs. Sárpo Mira, Maris Piper and Rywal, were tested for the presence of PVY using an ELISA 3 weeks post inoculation. The ELISA tests showed that all plants that yielded HR in the detached leaf test (cvs. Sárpo Mira, Rywal and 13 of the SM × MP progeny) were able to prevent PVY spreading to the upper leaves. In contrast, PVY was detected in the upper leaves of cv. Maris Piper and four of the SM × MP progeny that did not show an HR in the detached leaf assay, even though some apparently nonspecific necrosis reactions were observed in these progeny plants in whole plant assays (Table 2). The whole plant test was not only less specific but it was also less sensitive than the detached leaf assay, since HR was not detected in 13 resistant plants in this test. Taking together the results of both whole plant and detached leaf assays as well as the ELISA, we reproducibly identified 75 PVY-resistant and 65 susceptible individuals in the SM × MP progeny. This proportion is not significantly different from a 1:1 ratio (*χ* ^2^ = 0.714, *p* < 0.3978), and underlines the assumption that a single gene present in cv. Sárpo Mira in simplex underlies this trait. This dominant gene confers the PVY resistance that was manifested by the hypersensitivity response, and therefore we referred to it as *Ny*-*Smira* (Table 2).

**Table 2:** Phenotypic assessment of the presence of the *Ny*-*Smira* PVY resistance gene in parents and progeny of the SM × MP cross

Genotype	HR to PVY in		PVY (ELISA)	*Ny*-*Smira*
	Detached leaf test 2012	Whole plant test 2013		
Sárpo Mira	1	1	0	1
Maris Piper	0	0	1	0
Rywal (*Ny*-*1*)	1	1	0	na
Progeny (*N* = 4)	0	1	1	0
Progeny (*N* = 13)	1	0	0	1
Progeny (*N* = 61)	0	0	nt	0
Progeny (*N* = 62)	1	1	nt	1

1—Present, 0—absent

*nt* Not tested, *na* not applicable

**Fig. 2:**
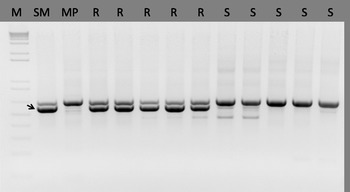
PCR amplification of marker Ry186 (Mori et al. 2011) on DNA templates of cvs. Sárpo Mira (*SM*) and Maris Piper (*MP*) and a sample of their PVY resistant (*R*) and susceptible (*S*) progeny. *Arrow* indicates marker allele band (588 bp) linked to the *Ny*-*Smira* gene; *M* 1-kb Plus DNA ladder, Invitrogen™ by Life Technologies

**Fig. 3:**
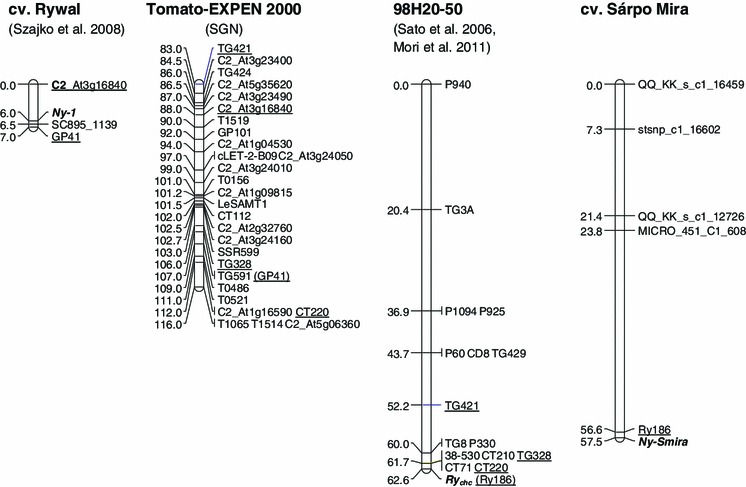
Locations of *Ny*-*1* (Szajko et al. 2007), *Ry*_*chc*_ (Sato et al. 2006; Mori et al. 2011) and *Ny*-*Smira* genes for PVY resistance on potato chromosome IX. Genetic linkage map of the cv. Sárpo Mira haplotype bearing the PVY resistance gene *Ny*-*Smira* was constructed on the basis of SM × MP progeny (Jupe 2012). A segment of Tomato-EXPEN 2000 map (SGN) is shown as a bridge between maps of cv. Rywal (Szajko et al. 2007) and 98H20-50 (Sato et al. 2006). To enable comparison, markers in *parentheses* GP41 and Ry186 are added on the basis of information from other sources: Tomato-EXPEN 1992 map (SGN) and Mori et al. (2011), respectively. Markers common between maps are underlined. On the *left* of each map, cumulative genetic distances are given in cM

To find the chromosomal localisation of the *Ny*-*Smira* gene, we tested markers linked to the *Ny* and *Ry* genes described previously (Flis et al. 2005; Song et al. 2005; Szajko et al. 2008, 2014). With the exception of the marker Ry186 which is linked to the *Ry*_*chc*_ gene located on potato chromosome IX (Sato et al. 2006; Mori et al. 2011), none of the other markers were linked to the *Ny*-*Smira* gene (data not shown). The Ry186 marker produced a polymorphic PCR product and segregated in the SM × MP progeny (Fig. 2). The nucleotide sequence of the marker band amplified from cv. Sárpo Mira (588 bp) was identical to the sequence amplified from the Japanese *Ry*_*chc*_ gene source plant (the Ry186 sequence was kindly provided by Professor Kazuyoshi Hosaka from the Potato Germplasm Enhancement Laboratory, Obihiro University of Agriculture and Veterinary Medicine, Japan). Within the 140 individuals of the SM × MP population, there were only two recombinants, indicating that Ry186 was located approximately 1.4 cM from the *Ny*-*Smira* gene. To confirm the localisation of the *Ny*-*Smira* gene on chromosome IX, we used marker data derived from a GoldenGate (Illumina) analysis (Jupe 2012). Since this genotyping data and PVY phenotyping data were available for a common set of 112 SM × MP progeny, we constructed a genetic map of a single linkage group harbouring the *Ny*-*Smira* gene (Fig. 3). For this purpose, simplex markers were extracted from the set of 383 polymorphic markers mapped in the SM × MP population (Jupe 2012) and tested for linkage with PVY resistance. Only four GoldenGate markers were linked in coupling with the *Ny*-*Smira* gene, but they could all be positioned on chromosome IX (Jupe 2012).

**Fig. 4:**
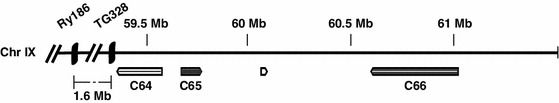
Representation of the NB-LRR hot spot on chromosome IX. *Ny*-*Smira* has been mapped to the long arm of chromosome IX, distal from marker Ry186, in front of a NB-LRR resistance gene hot spot. Three main clusters were identified within the potato reference genome (C64–C66), and several Solanaceae *R* genes were cloned from this region, including ones conferring resistance to the late blight (*Rpi*-*vnt1.1*) and the viruses (*Tm*-*2*, *Sw*-*5*)

The linked marker Ry186 localised to position 57,765,683 (Fig. 4). It resided proximal to the NB-LRR gene cluster C64 on reference chromosome IX of the sequenced potato clone DM (Jupe et al. 2013). The first NB-LRR gene within cluster C64 is RDC0001NLR0219 at position 59,375,661 and a further 18 CNL genes with sequence similarity to the late blight resistance gene *Rpi*-*vnt1* and the virus resistance gene *Tm*-*2* were found within this region (Foster et al. 2009; Lanfermeijer et al. 2003). Approximately 105 kb towards the end of chromosome IX is the homogenous TNL cluster C65, and starting at position 60,607,803 is a further cluster, C66, that harbours sequences similar to *Sw*-*5* (Brommonschenkel et al. 2000) (Fig. 4).

## Discussion

In this study we were able to show that potato cultivar Sárpo Mira is resistant to PVY, confirming earlier reports (The European Cultivated Potato Database). Here we report for the first time that the resistance is associated with the hypersensitive response which is elicited at both 20 and 28 °C. This response resembles the resistance response of cv. Romula containing the PVY resistance gene *Ny*-*2* (Szajko et al. 2014). HR in cv. Sárpo Mira was observed in whole plant and detached leaf assays. ELISA tests further demonstrated that in a whole plant experiment after 3 weeks at 20 °C, PVY did not spread systemically to uninoculated leaves of cv. Sárpo Mira. The PVY isolate 12–94 used in our study is a representative of the PVY^NTN^ strain that is especially harmful for potato tuber quality.

The segregation of the HR to PVY infection was tested in 140 individuals of SM × MP progeny in both detached leaf and whole plant assays. The results indicate that a single, dominant gene in simplex was underlying this trait in cv. Sárpo Mira. The gene, *Ny*-*Smira*, was then mapped using the sequence-specific PCR marker Ry186 (Mori et al. 2011) and GoldenGate assay markers (Illumina, Jupe 2012) to potato chromosome IX. The resistance corresponded to the map position of the previously identified gene *Ry*_*chc*_ (Fig. 3) from *S. chacoense* for extreme resistance to PVY (Hosaka et al. 2001; Sato et al. 2006). The marker Ry186, which is located 1.4 cM from the *Ny*-*Smira* gene, is also in close proximity (0.203 cM) to the *Ry*_*chc*_ gene (Mori et al. 2011). This marker, as well as the position of the four GoldenGate assay markers, locates *Ny*-*Smira* on chromosome IX. Using the *Ry*_*chc*_-associated markers Ry364 (59.4 Mb), TG328 (59.4 Mb) and 38-510 (61.1 Mb), we were able to anchor *Ny*-*Smira* to the long arm. The tetraploid character of the underlying mapping population made it difficult to identify additional, already mapped, markers which would allow a more precise localisation. We used the Tomato-EXPEN 2000 (SGN) map of chromosome IX, which shared some markers with both the map of 98H20-50 containing *Ry*_*chc*_ (Sato et al. 2006; Mori et al. 2011) and the map of cv. Rywal with the *Ny*-*1* gene (Szajko et al. 2008), to compare their locations (Fig. 3). This comparison shows that, on the basis of genetic and genomic mapping, it is likely either that these genes are from the same *R* gene cluster harbouring NB-LRR encoding genes, or that they are different alleles occupying the same locus.

The location of the *Ny*-*Smira* gene is within the proximity of a resistance gene hotspot that contains 46 NB-LRR genes, organised in clusters C64 to C66 in the DM potato reference genome (Jupe et al. 2013). Cluster C64 harbours both TNL and CNL genes and, amongst genes related to the late blight resistance gene *Rpi*-*vnt1*, contains genes with homology to the tomato *Tm*-*2* gene that provides resistance to the tomato mosaic virus (Lanfermeijer et al. 2003).

When comparing different genetic maps, in addition to PVY resistance genes, another virus resistance gene originating from *S. phureja*, *Nx*_*phu*_, which confers a HR-based resistance to *Potato virus X* (PVX) (Tommiska et al. 1998), can be located within the terminal part of chromosome IX. The *Sw*-*5* locus providing resistance to tomato spotted wilt tospovirus (TSWV) has been identified in a syntenic chromosomal position of the wild tomato species *S. peruvianum* (Stevens et al. 1995; Brommonschenkel and Tanksley 1997). At least five genes for resistance to *P. infestans* have been mapped to the same region and include *Rpi*-*mcq1* from *S. mochiquense* (Smilde et al. 2005), *Rpi*-*edn2* from *S. edinense* (Verzaux 2010), *Rpi*-*dlc1* from *S. dulcamara* (Golas et al. 2010), *R8* from *S. demissum* (Jo et al. 2011) and *Ph*-*3* from *S. pimpinellifolium* (Chunwongse et al. 2002).

While the origin of the *Ry*_*chc*_ gene from *S. chacoense* is well documented (Hosaka et al. 2001), little is known about the sources of *Ny*-*1* and *Ny*-*Smira*. In the pedigree of Polish cv. Rywal, in which *Ny*-*1* was identified, *S. chacoense* cannot be found. However, as some of its ancestors remain elusive, the possibility that this species was among them cannot be excluded. In the case of cv. Sárpo Mira, the pedigree data is limited to only one generation, 76.PO.12.14.268 × D187, and no information on the resistance source is available (www: Potato Pedigree Database). *S. chacoense* has been described as a source of not only the *Ry*_*chc*_ gene but also *Ny*_*chc*_ which could be activated by all strains of PVY available at that time (Cockerham 1970). Previously, the PVY extreme resistance genes were thought to be clearly distinct from the *Ny* genes, which in comparison to no symptoms develop necrotic lesions at the site of infection (Valkonen et al. 1996). Later, it was shown that both resistances rely on HRs that, however, develop at different speeds (Bendahmane et al. 1999; Celebi-Toprak et al. 2002). Our data add genetic evidence that the *Ry* and *Ny* genes can be found in the same genomic region and that they may be alleles originating from different wild potato species. Vidal et al. (2002) provided some evidence for one more possible explanation: that the genetic background of the host plant can affect the resistance reaction, resulting in necroses in some genotypes. Evidence for this has been provided by the experiment in which a NB-LRR DNA sequence *Y*-*1* cosegregating with the *Ry*_*adg*_ gene has been expressed in transgenic potato under the control of *Cauliflower mosaic virus* 35S promoter. These plants exhibited necrotic lesions after infection with PVY but they were systemically infected with the virus. The authors have not excluded the possibility that *Y*-*1* could be *Ry*_*adg*_, but they have rather considered these two genes to be different but tightly linked. Another explanation could be that *Y*-*1*, being in fact *Ry*_*adg*_ in a different genetic background and under the control of a different promoter, conferred the necrotic reaction, perhaps due to the variation in interaction between the R protein and its downstream signaling components (Vidal et al. 2002).

The *Ry*_*sto*_ gene provides high levels of resistance to PVY in a number of potato cultivars released from various breeding programs worldwide which have proved to be durable for over 30 years (Flis et al. 2005; Song et al. 2005; Dalla Rizza et al. 2006; Valkonen et al. 2008; Ottoman et al. 2009; Zimnoch-Guzowska et al. 2013). However, a monogenic resistance is, more than other resistance types, threatened by the possible evolution of new, mutated pathogen strains that overcome the single resistance. Therefore, exploiting many resistance sources and genes seems to be a better and safer strategy that will lower selection pressure on pathogen population. One such gene for resistance to PVY could be *Ny*-*Smira,* found in cv. Sárpo Mira. Since this cultivar is currently being used by potato breeders, knowledge of the linkage of the marker Ry186 to the *Ny*-*Smira* gene can be applied in breeding programs to facilitate selection of the PVY-resistant lines.
